# Evidence of widespread pollen limitation in diverse specialty crops on commercial farms

**DOI:** 10.1098/rsos.250201

**Published:** 2025-04-09

**Authors:** Eleanor Stroh, Ashley Leach, Zeus Mateos-Fierro, Ian Kaplan

**Affiliations:** ^1^ Department of Entomology, Purdue University, West Lafayette, IN, USA; ^2^ Department of Entomology, The Ohio State University, Wooster, OH, USA

**Keywords:** pollination services, crop yield, tomato, apple, blueberry, watermelon

## Abstract

Specialty crops (non-staple fruits and vegetables) have diverse pollination requirements and pollinator communities, yet production may be limited by pollen limitation. We quantified insect pollination and pollen limitation on commercial farms across crops that differ in their reliance on insect pollination, including watermelons, apples, blueberries and tomatoes. We also considered the impact of thinning in apples and protected culture of tomatoes on pollination dynamics. We compared fruit set and quality across insect exclusion, open pollination and hand pollination treatments. Insect pollination increased fruit set in every crop (increase from exclusion to open treatments; mean = 22.9%, range = 1.1–48.0%), while hand pollination increased fruit set across crops, except for apples after thinning (increase from open to hand pollination treatments; mean = 5.8%, range = 0.3–8.7%). The effect of pollination treatment on fruit quality varied. Field tomatoes were the only crop to demonstrate pollen limitation in all metrics. Despite evidence for ambient pollinator contributions across specialty crops, our work highlights the opportunity for further increases in yield, particularly in crops not considered dependent on pollinators like tomatoes.

## 1. Introduction

Insects are essential pollinators of fruits and vegetables [1]. Recent declines in pollinator populations, coupled with increased cultivation of pollinator-dependent crops, have led to growing concern about losses of pollination services in diverse crops [2,3]. The term for this phenomenon, ‘pollen limitation’, describes a scenario where a lack of adequate pollen deposition to flowers results in submaximal crop yields. Pollen limitation is caused by low pollinator abundance or flower visitation rates [4] and is reflected in a positive relationship between pollinator visitation and yield. Crops vary in the extent to which they experience pollen limitation. In a meta-analysis across 108 crops, supplemental pollination increased fruit set by an average of 27% relative to an insect pollination control, but values ranged from 0 to 100% depending on the crop [5]. For example, apples (*Malus domestica*) experience a maximum of 70% increase in fruit set with supplemental hand pollination [6] and a mean increase in yield of 167% [7]. For blueberries (*Vaccinium corymbosum*), the maximum increase in fruit set is approximately 5% [8], with increases in yield ranging from 12 to 23% [9]. Meanwhile, 0 and 10% of watermelons (*Citrullus lanatus*) are pollen-limited in Florida and California, respectively [10], and supplemental pollination does not increase fruit set for tomatoes (*Solanum lycopersicum*) [11,12]. These data indicate that pollen limitation varies between crops, but the crop-specific factors driving variable pollen limitation are unclear.

Pollen limitation may be influenced by pollinator dependence, which relates to flower morphology and breeding system. For example, hermaphroditic and self-compatible crops (e.g. tomatoes) are classified as less pollinator-dependent than hermaphroditic but (variably) self-incompatible crops like apples and blueberries [1]. Meanwhile, monoecious and self-incompatible crops (e.g. watermelon) always require cross-pollination from a pollenizer cultivar, necessitating insect visitation [13]. Pollinator-dependent crops may be more likely to experience pollen limitation due to their reliance on insect visitation. However, this is accounted for in many crops by use of managed bees (e.g. western honeybees, *Apis mellifera*) to ensure consistent pollination and secure commercial yield [14]. This practice may be sufficient to alleviate pollen limitation, as evidenced by the low frequency of pollen limitation in watermelon and almonds (*Prunus dulcis*) [10]. Yet, despite the use of managed bees, apples and blueberries typically experience pollen limitation [6,10,15]. One meta-analysis demonstrated that use of honeybees decreases pollen limitation in self-compatible but not in self-incompatible crops [16]. Thus, degree of pollinator dependence does not necessarily correlate to severity or frequency of pollen limitation, nor is the use of managed bees always sufficient to eliminate it. Comparison of pollination services across multiple crops in a single production region and concurrent years would elucidate trends in pollen limitation and their relation to pollinator dependence and management.

Aside from the use of commercial pollinators, other crop management practices also affect yield metrics (e.g. fruit set and weight), with implications for pollination outcomes [8,11,17–19]. In apple production, for instance, growers almost always thin trees after bloom to reduce the number of developing fruits, resulting in larger apples that have greater consumer appeal [20]. As a result, the frequency and magnitude of pollen limitation may vary depending on when in the season it is measured (i.e. pre- vs. post-thinning). Similarly, the use of protected culture to extend the growing season, such as growing under plastic-covered high tunnels, may indirectly shape pollination dynamics [21,22]. Difficulty entering and navigating in the tunnels [22,23], combined with high summer temperatures [21], may decrease insect visitation and pollination services compared with crops grown outdoors. Despite their increasing popularity, few studies have investigated pollination dynamics in high tunnels (but see [22]) particularly for crops like tomatoes that are not considered pollinator-dependent.

We evaluated pollination and pollen limitation in watermelons, apples, blueberries and two types of tomatoes—those grown in high tunnels and open fields. The study was conducted in Indiana, USA, a novel landscape for quantifying pollen limitation across diverse cropping systems. Even though specialty crop production in Indiana is isolated in a simplified agricultural landscape dominated by corn (*Zea mays*) and soybean (*Glycine max*), flower visitation rates and pollinator community composition differ between specialty crops, suggesting variation in the pollination services they receive [24]. Pollination was experimentally manipulated on commercial farms over three years to quantify the impact of insect pollination and pollen limitation on fruit set and quality. The aims of our study were to (i) identify crop systems experiencing pollen limitation and compare the extent to which they qualitatively differ and (ii) investigate the potential role of thinning in apples and protected culture in tomatoes in influencing pollen limitation.

## 2. Material and methods

### 2.1. Crop systems

We intentionally chose a variety of specialty crops that vary in their pollinator needs, focusing on (from most to least pollinator-dependent) watermelon, apple, blueberry and tomato [1]. Pollinator dependence values between 0 and 1 reflect the extent to which each crop relies on animal pollination: watermelon (0.87), apples (0.73), blueberries (0.53) and tomatoes (0.27) [16]. Field-grown tomatoes and watermelons have the largest specialty crop acreage in Indiana, where annual production area totals 2867 and 2792 ha, respectively [25]. Both are grown in large fields (generally greater than 20 ha) and bloom in the summer. Watermelons are sold fresh, while tomatoes are grown for processing into tomato-based products. Because pollination is an essential component of watermelon production, fields are stocked with up to two species of managed bees; all growers supplement with honeybees (1 hive/acre) and approximately half also supplement with Common Eastern bumblebees (*Bombus impatiens*) (0.5−1 hive/acre) (author’s personal observation). Conversely, tomatoes are not stocked with managed bees in field settings.

Smaller-scale operations that grow a diverse array of crops are also common in Indiana. Many diversified farms use high tunnels to extend the growing season, such that crops like tomatoes can be planted earlier in the spring. In 2022, 260 farms in Indiana grew tomatoes ‘under protection’ in greenhouses and high tunnels, totalling greater than 1 million square feet of production (approx. 10 ha) [25]. Although growers occasionally stock high tunnels with bumblebees (approx. 1 hive/high tunnel) to counteract low wind, this is not a widespread practice (author’s personal observation). With 397 apple orchards and 165 blueberry farms in the state [25], apple and blueberry growers are some of the most numerous farms in Indiana operating on a small scale. Apples and blueberries bloom in the spring and are stocked with managed honeybees to ensure consistent yields. Indiana growers follow conventional recommendations of 1−2 hives/acre in apples and 2−3 hives/acre in blueberries [14].

### 2.2. Farm selection

A total of 30 commercial farms were recruited to participate in this three-year study. In 2022, we recruited five apple and five blueberry farms, three high tunnel and three field tomato farms and two watermelon farms. In 2023 and 2024, we recruited five farms for each crop. In several crops, 1−2 farms were replaced between years, but otherwise, farms stayed the same between years. A ‘farm’ consisted of an individual grower. In the case of apples, blueberries and high tunnels, all production was centralized in a single locality, which did not change year to year; thus, the same area was sampled over multiple years. Field tomatoes and watermelons are rotated with corn and soybean on an annual basis; each year, we selected a single field for sampling from each farm, and the sampling locations changed year to year. To account for low farm recruitment for watermelons in 2022, three separate fields from one of the farms were selected.

Throughout the paper, we use ‘field’ to refer to the sampled area, as it is the most inclusive term across crops. Fields were each greater than 2.5 km apart, except for one instance in watermelon, where the minimum between-field distance was 1.3 km. The entire cultivated area on apple and blueberry farms averaged 12.3 and 7.0 ha, respectively. The individual fields for field tomatoes and watermelons averaged 39.2 and 30.2 ha, respectively. Individual high tunnels (one selected per farm) averaged 0.03 ha in size. All fields were non-organic and conventionally managed. Participation in the study did not alter management practices and therefore grower inputs (e.g. pesticide use or pollinator management) are considered representative of the region.

Cultivars were selected based on their commonality between farms, which was often tied to grower and consumer preference. Gala apples were initially selected in 2022, but due to changes in farm participation, we switched to Honeycrisp in 2023 and 2024. Blue-ray blueberries were used for all three years. Red slicer tomatoes were selected in high tunnels, with some cultivar differences between fields (the most common was Red Deuce). TSH−4 var. tomatoes were used for field tomatoes. Large, seedless cultivars were selected in watermelons, although high variability in preference between growers caused cultivars to differ between fields (electronic supplementary material, table S1).

### 2.3. Pollination experiment

Two transects were established within the selected cultivar in each field, along which experimental pollination treatments (hereafter, pollination treatments) were applied. One transect was located 5−10 m from and parallel to the edge of the planted area and the other near the centre, to account for potential differences in pollination services between edge and interior [26]. Transects in field tomatoes and watermelons were 100 m long and ran along a crop row. They were positioned such that the centre of the transect aligned with the centre of the crop row. Due to variability in the length of crop rows in apples, blueberries and high tunnel tomatoes, transects spanned the entire length of the row, averaging 119, 102 and 40 m, respectively. To compensate for short row lengths in high tunnels, three transects were established in each high tunnel along three randomly selected rows. The tunnels were small enough (less than 15 m diameter) that they lacked distinct ‘edge’ and ‘centre’ areas, so transects were not selected with location in mind.

We subjected flowers in each transect to three pollination treatments: (i) pollinator exclusion, (ii) supplemental hand pollination (hereafter, ‘hand pollination’), and (iii) uncovered, unmanipulated flowers (hereafter, ‘open pollination’). In the pollinator exclusion treatment, flowers were covered with a mesh 1-gallon paint strainer bag (Trimaco^®^ SuperTuff^®^ item no. 11311/25) prior to anthesis, with bags enclosing the unopened flower buds and secured using twist ties to the branch/plant stem (electronic supplementary material, figure S1). The mesh was fine enough (600 µm) to prevent insect visitation but allowed pollination by wind. Bags were removed after flowers were senesced. In the open pollination treatment, flowers were left open for insect pollination, providing an estimate of current pollination services. In the hand pollination treatment, flowers were pollinated manually, in addition to being left open for insect pollination, to simulate a maximum pollination scenario.

Flowers in each pollination treatment replicate were counted and marked before bloom. For apples and blueberries, each replicate consisted of two−four consecutive flower clusters, totalling approximately 20 flowers per replicate. Five trees/bushes were randomly selected in each transect and two replicates of each treatment were established on separate branches of each tree/bush (a total of 20 replicates/treatment/field). For both types of tomatoes, replicates consisted of a single inflorescence on a plant, each containing five−eight flowers. For watermelons, each replicate consisted of a single female flower. In the case of field tomatoes and watermelons, treatments were randomly assigned (one each) to 30 individual plants spaced 3 m apart along the transect. Ten replicates of each treatment were established along each transect, again totalling 20 replicates/treatment/field. Due to limited plant numbers in some high tunnels, only five replicates of each treatment were established along 15 randomly selected plants in each transect, totalling 15 replicates/treatment in each high tunnel.

In apples and blueberries, flowers were cross-pollinated with pollen collected from a nearby co-blooming compatible cultivar. Pollen was applied to stigmas with a paintbrush, using methodology described in Garratt *et al*. [19] (electronic supplementary material, figure S1). Hand pollination was performed once a day on four separate days over the course of the bloom period. Due to variation in non-study cultivars between fields, different outcross pollen was used in different fields (electronic supplementary material, table S2). In 2022, because the self-compatibility of Blue-ray was unknown, an additional self-pollination treatment was implemented in blueberries with pollen collected from the same bush as the experimental treatments. This treatment was discontinued in subsequent years after data analysis indicated that there was no difference in yield or quality metrics between the self-pollination and cross-pollination treatments (electronic supplementary material, figure S2), and in subsequent analyses, replicates from both treatments were combined under the same level of ‘hand pollination’.

Tomato flowers, which are self-compatible in most cultivars including the cultivar used in this study [27], were self-pollinated using a vibrating VegiBee^tm^ tomato wand. The wand tip was placed near the distal end of the anther cone for approximately 4 s per flower. The vibrations mimic the sonication provided by buzz-pollinating bees, which move the pollen from the anther cone onto the stigma and increase deposition of self-pollen [28]. Watermelon flowers were cross-pollinated by hand using male flowers of the pollinizer cultivar available in the field (electronic supplementary material, table S1). Male flowers were harvested on the morning that hand pollination occurred, and their anthers were lightly brushed over the stigma of a female flower. For each female flower, eight male flowers were used to ensure adequate pollination.

### 2.4. Yield metrics

The fruit set was calculated as the percentage of flowers in each replicate that produced fruit. Fruit set was quantified at, or just before, harvest. An additional count of fruit set in apples took place prior to chemical thinning (approx. one week after bloom end). Fruits were harvested as they ripened or just prior to commercial harvest. Any unripe fruits were included in counts of fruit sets but excluded from other measurements.

Apples, watermelons and high-tunnel tomatoes were weighed individually. Field tomatoes and blueberries from a single replicate were weighed together and divided by the number of fruits to obtain an average fruit weight. In 2024, several transects of personal-sized watermelons were accidentally selected for the experiment. Due to low fruit sets across all fields, the removal of these data points reduced the 2024 watermelon weight dataset to less than five replicates per treatment; consequently, this year was omitted from weight analysis.

Sugar content was quantified in each crop using a handheld electronic refractometer (Atago Pal−1, item no. 3810), which measures Brix (expressed as per cent total soluble solids), a proxy for sugar content. Fruits at room temperature (approx. 20°C) were crushed in a mortar and pestle or blended with an immersion blender to produce juice, which was pipetted into the refractometer. Brix was measured individually for each apple, high tunnel tomato and watermelon, while blueberries and field tomatoes in each replicate were combined to yield sufficient juice for measurement. Three measurements were taken in each watermelon, with samples taken from the edge of the ripe flesh, the centre and in between. Samples were averaged to obtain a single Brix measurement for each watermelon. Sugar content was measured in 2022 and 2023 for all crops except field tomatoes, for which it was measured in 2023 and 2024.

Several additional quality measurements were taken in individual crops. In apples, we measured maximum diameter; apples with a diameter less than 2.5 in (6.3 cm) are considered unmarketable for fresh sale [29], although many growers in this study sold smaller apples at their local farm stand. A roundness index was calculated for each high tunnel tomato using its minimum and maximum diameter, following the equation given by Morandin *et al*. [30]. Roundness in tomatoes contributes to consumer appeal and may be correlated with increased pollination [31]. Finally, in watermelon, the presence or absence of hollow heart disease was noted for each fruit. Watermelons were initially scored on a 1−5 scale for severity of tissue separation [32], but due to low incidence of hollow heart, data were translated into presence (score of 2−5) or absence (score of 1). Hollow heart is related to suboptimal pollination and results in separation of heart tissue [32,33].

### 2.5. Statistical analyses

All analyses were conducted in R v. 4.4.0 [34], using packages lme4 and glmmTMB for statistical analysis [35] and ggplot2 for data visualization [36,37]. Due to differences in sampling design, model structure for each crop and yield metric was variable, although fixed effect structure remained the same (electronic supplementary material, table S3). Pollination treatment and sampling year were included as fixed effects; the latter to investigate potential environmental differences that might affect pollen limitation. Random effects included the nested variables field, transect, tree/bush and replicate (where applicable). In crops where sampling year was partly confounded with field (e.g. where fields were added or lost in particular years), a combined ‘fieldyear’ variable was included in place of ‘field’ as a random effect; ‘year’ was retained as a separate fixed effect. In several instances the random effect structure was simplified to account for singular solutions in the maximally complex models (electronic supplementary material, table S3). Because we switched apple varieties between 2022 and 2023, sampling year and apple cultivar were partially confounded. Thus, to account for pollen limitation differences between Gala and Honeycrisp [38], we fit separate models to avoid the confounding effect of cultivars.

The effects of pollination treatment on fruit set (ratio of fruit set to number of flowers), apple diameter (above or below the marketable threshold) and watermelon hollow heart (presence/absence) were analysed using generalized linear mixed models with a binomial distribution and a log link function. A beta-binomial error structure was used to correct for overdispersion when it was detected in binomial models. Low fruit set in the pollinator exclusion treatment for apples and watermelons resulted in poor replication for this treatment level, and the level was dropped from subsequent analyses of quality metrics. The effects of pollination treatment on fruit weight, sugar content (Brix) and tomato roundness were analysed using linear mixed models. In several models, variables were square root or log transformed to correct for non-normality of raw data (electronic supplementary material, table S3). We used type II ANOVA to identify significant model terms and Tukey post hoc tests (with package ‘multcomp’; [39]) for pairwise comparisons between pollination treatment levels and years.

## 3. Results

### 3.1. Fruit set

**Figure 1.**
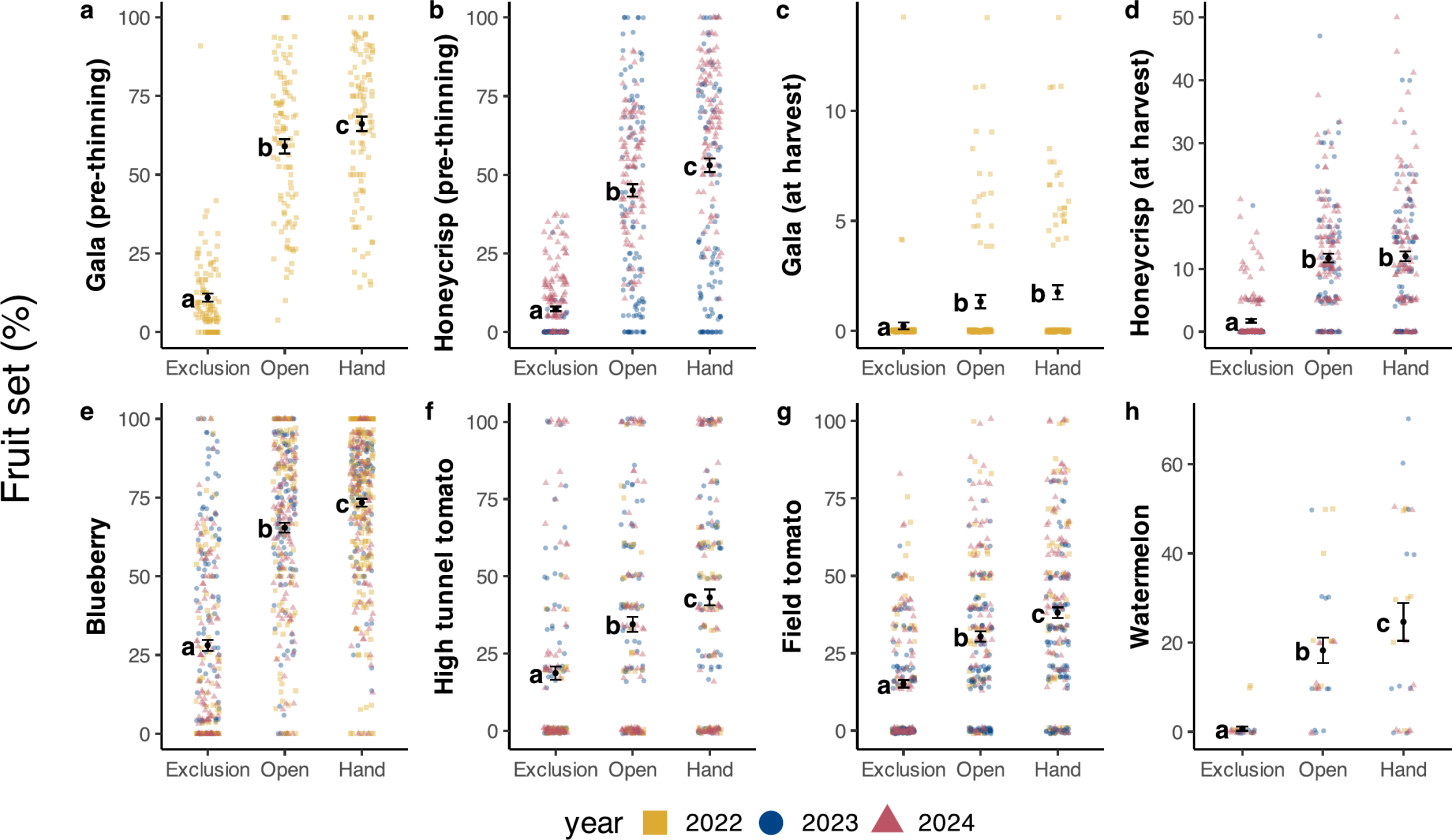
Mean ± s.e. fruit set (%) in each pollination treatment and crop system. Panels a–d display apple fruit set pre-thinning (a,b) and post-thinning (c,d) for Galas (a,c) and Honeycrisp (b,d). Data points represent the fruit set in each replicate, and are shape and colour coded by year. In the watermelon panel (h), data points represent the average fruit set along each transect in each field.

Pollination treatment affected fruit sets in all crop systems (table 1). Fruit set in the exclusion treatment was very low for watermelons and apples (figure 1). Fruit set significantly increased from the pollinator exclusion to the open pollination treatment in each crop (figure 1); however, crops varied in the magnitude of their response to insect pollination, as determined by the absolute increase in fruit set from pollinator exclusion to open pollination. Apple responses were the greatest initially, with increases of +48.0% (Gala) and +37.7% (Honeycrisp), followed by blueberries (+37.3%), watermelons (+17.6%), high tunnel tomatoes (+15.9%) and field tomatoes (+15.3%) (electronic supplementary material, table S4). After thinning, however, apple increases were the lowest (+10.0% for Honeycrisp and +1.1% for Gala) (figure 1, electronic supplementary material, table S4). Fruit sets significantly differed between years in blueberries, tomatoes and apples (initial fruit set), but not other crops (table 1, electronic supplementary material, table S5).

**Table 1.** Results of fruit set and weight models for each crop. In apples, ‘initial’ refers to fruit set pre-thinning and ‘final’ refers to fruit set at harvest. The year was not included in Gala models because there was only one year of data for that cultivar. In apple and watermelon fruit weight models, there were only two treatment levels (open and hand pollination) included in analysis. Asterisks indicate level of significance for *p*-values: no asterisk for *p* ≥ 0.05, * *p* = 0.05–0.01, ** *p* = 0.01–0.001, and *** *p* ≤0.001.

yield metric	crop	variable	*Χ* ^*2*^	*df*	*p*‐value
fruit set	Gala: initial	treatment	509.36	2	<0.001***
	Honeycrisp: initial	treatment	622.64	2	<0.001***
		year	4.63	1	0.031*
	Gala: final	treatment	15.13	2	0.001**
	Honeycrisp: final	treatment	231.28	2	<0.001***
		year	2.49	1	0.115
	blueberry	treatment	479.72	2	<0.001***
		year	17.76	2	<0.001***
	high tunnel tomato	treatment	160.12	2	<0.001***
		year	84.69	2	<0.001***
	field tomato	treatment	178.03	2	<0.001***
		year	8.54	2	0.014*
	watermelon	treatment	32.08	2	<0.001***
		year	3.87	2	0.144
fruit weight	Gala	treatment	0.15	1	0.701
	Honeycrisp	treatment	4.21	1	0.040*
		year	5.15	1	0.023*
	blueberry	treatment	109.48	2	<0.001***
		year	42.62	2	<0.001***
	high tunnel tomato	treatment	21.83	2	<0.001***
		year	5.88	2	0.053
	field tomato	treatment	67.80	2	<0.001***
		year	0.41	2	0.815
	watermelon	treatment	0.06	1	0.809
		year	0.58	1	0.447

Fruit set significantly increased from open pollination to the hand pollination treatment in every crop except for apples at harvest (figure 1). The extent of pollen limitation varied between crops, determined by the absolute increase in fruit set from open to hand pollination. High tunnel tomatoes had the largest absolute increase (+8.7%), followed by blueberries and Honeycrisp (initial) (both +8.0%), field tomatoes (+7.7%), Gala (initial) (+7.1%) and watermelons (+6.4%) (electronic supplementary material, table S4).

### 3.2. Fruit weight

**Figure 2.**
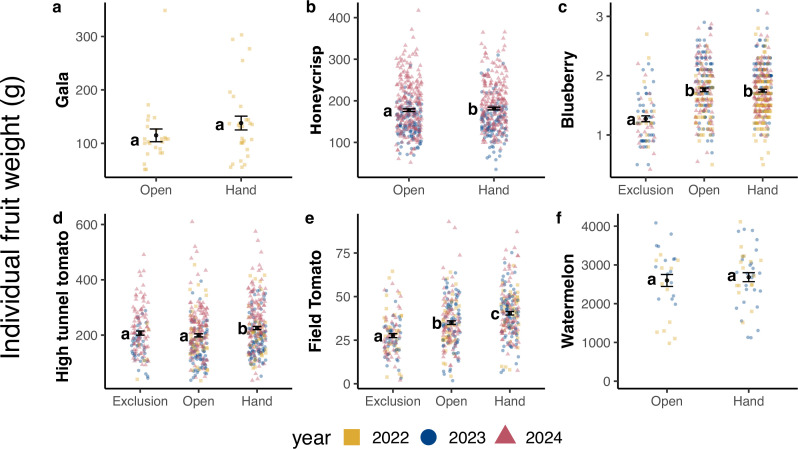
Mean ± s.e. individual fruit weight in each pollination treatment and crop system. Data points in panels a, b, d and f represent weights of individual fruits. Because blueberries and field tomatoes were not weighed individually, data points in panels c and e represent mean fruit weight per replicate. Only two treatment levels (open and hand pollination) are included for apples and watermelon due to low yield in the exclusion treatment; watermelon data from 2024 are excluded for the same reason.

Individual fruit weight significantly varied between pollination treatments in Honeycrisp apples, blueberries and both tomatoes (table 1). For the crops that yielded enough fruit in the pollinator exclusion treatment for analysis, there was variability in the relative increase in fruit weight from pollinator exclusion to open pollination. Blueberry weight significantly increased by 27.8% with insect pollination (electronic supplementary material, table S4). While there was no evidence that open pollination increased high tunnel tomato weight, there was a 20.2% increase in field tomato weight from pollinator exclusion to hand pollination (figure 2, electronic supplementary material, table S4). Fruit weight varied significantly between years in apples and blueberries, but not other crops (table 1, electronic supplementary material, table S5).

The extent of pollen limitation in fruit weight varied between crops, determined by the relative increase in fruit weight from open to hand pollination. Fruit weight significantly increased from open to hand pollination in field tomatoes (+13.4%), high tunnel tomatoes (+11.7%) and Honeycrisp (+2.1%) and although the largest relative increase in fruit weight was observed in Gala (+16.7%), this difference was not statistically significant (table 1, electronic supplementary material, table S4). Additionally, there was little evidence that watermelons or blueberries varied in weight between open and hand pollination treatments (table 1, figure 2).

### 3.3. Other quality metrics

Several other metrics relating to fruit quality displayed evidence of a relationship with pollination treatment. Although sugar content did not vary between pollination treatments or for apples, high tunnel tomatoes, blueberries or watermelons, in field tomatoes the relative increase of 6.0% from exclusion to open pollination and 3.3% from open to hand pollination was significant (electronic supplementary material, table S6, figure S3). Sugar content also varied between years in field tomatoes and blueberries, but not other crops (electronic supplementary material, table S6). Additionally, Honeycrisp diameter was significantly (+3.0%) larger in the hand pollination treatment than the open pollination treatment, and diameter differed between years, being greatest in 2024 (electronic supplementary material, table S6, figure S4). Although Gala diameter was 4.0% larger in hand versus open-pollinated apples, this difference was not significant (electronic supplementary material, table S6, figure S4). Tomato roundness in high tunnel tomatoes and incidence of hollow heart in watermelon showed no evidence of a relationship to pollination treatments or variation between years (electronic supplementary material, table S6).

## 4. Discussion

Our results indicate that insect pollination and pollen limitation partially correlate to degree of pollinator dependence. Insect visitation increased fruit set relative to the pollinator exclusion treatment in every cropping system, and supplemental pollen further increased fruit set except at harvest in apples, implying that thinning counteracts initial pollen limitation. The effect of insect visitation and supplemental pollination on fruit weight was variable between crop systems. We did not find strong evidence for pollen limitation on fruit weight in several pollinator-dependent crops, including blueberries and watermelons, but we did for both tomatoes. Insect visitation in high tunnel tomatoes, however, did not increase tomato weight relative to insect exclusion. Interestingly, tomatoes—the system least dependent on pollinators—showed evidence of pollen limitation in more yield and quality metrics than other crops.

### 4.1. Apple pollen limitation and thinning

The effect of pollination treatment on apple yield metrics at harvest offers compelling evidence that thinning partially mitigates initial pollen limitation in apples. All growers in the study thinned their apples, so we did not evaluate the effect of thinning, *per se*. However, pre- and post-thinning fruit set counts give an indication of the effect of thinning on pollen limitation; while hand pollination improved fruit set prior to thinning, fruit set did not differ between open and hand-pollinated treatments at harvest in either cultivar. Some other studies also report no difference in fruit set between open and hand-pollinated treatments at harvest [40]. Recent investigations into the potential of using insect netting during bloom to intentionally reduce insect visitation indicate that such a practice can negate the need to thin apples [41]. If this is the case, then the use of managed honeybees to supplement apple pollination may function best as a form of insurance for achieving consistent yields, rather than as a crucial component of apple production. Scaling down the use of honeybees could simultaneously decrease the need for extensive thinning, reducing costs overall for apple growers.

While thinning negated pollen limitation in apple fruit set, both varieties demonstrated a trend towards increased weight and diameter in the hand-pollinated treatment. Although these trends were not significant for Gala apples, this may be the result of a small dataset. Many growers overthinned their apples in 2022 (when Gala was sampled), resulting in low replication. Other studies show 9% reductions in weight and 4% reductions in diameter in open-pollinated Galas relative to hand pollination [42]. Similarly, in our study, modest improvements in apple size—particularly in 2022— resulted in a greater number of hand-pollinated apples falling above the threshold of commercial marketability per United States Department of Agriculture (USDA) size standards [29]. However, many of the small-scale producers in our study primarily sold apples direct to consumers (e.g. at farmers markets, farm stands and through ‘you-pick’ operations), and often sold small apples below the ‘marketable’ threshold. Thus, although Indiana apples were somewhat pollen-limited with respect to size, this may be a less consequential metric for small-scale producers that are not beholden to strict marketability standards.

### 4.2. Pollen limitation in blueberries and watermelon

We found that self-compatible blueberries still benefitted from pollination services, as fruit set and weight improved with insect pollination. Hand pollination further improved fruit set by 8%, in line with trends seen in other blueberry systems, where fruit set was improved by 1–10% [9]. However, blueberry weight was not pollen-limited. Other cultivars that are known to be self-compatible (e.g. Duke) also experience no increase in weight with supplemental pollination [43]. Our preliminary research in 2022 demonstrated that, like Duke, Blue-ray is a self-compatible cultivar. This highlights the importance of self-compatibility in shaping pollen limitation between blueberry cultivars, adding insight for a regionally popular cultivar in smaller-scale production for the Midwestern United States.

Watermelons demonstrated the strongest difference in fruit set between pollinator exclusion and open pollination of any crop, but we only found evidence for pollen limitation in fruit set. The low fruit set with insect exclusion shows the heavy pollinator dependence of watermelon; across three years, one bagged flower produced a watermelon, which could be attributed to experimental error. The low fruit set in the other treatments resulted in a small dataset and low predictive power for analyses of weight and hollow heart. This was compounded by the fact that we were unable to analyse weight data for 2024. Thus, the results of the watermelon analyses should be interpreted with caution. Even though the hand-pollination treatment had a 6.4% higher fruit set than open pollination, hand-pollinated watermelons showed little evidence of increase in weight (increase of 0.18 kg). Other studies in Indiana have shown no effect of supplemental pollination on watermelon weight (mean increase of less than 1 kg), but they also indicated no difference in fruit set [44]. In other regions, evidence for pollen limitation is also irregular [10], suggesting that watermelons are particularly variable in their responses to supplemental pollination.

### 4.3. Tomato pollination and protected culture

High tunnel and field tomato fruit sets responded similarly to pollination treatments. Fruit sets of high tunnel and field-grown tomatoes benefitted from insect visitation (an increase of 15.9 and 15.3%, respectively), which fits within the 5–32% range reported from studies on field tomatoes in Brazil [45,46]. Both tomato systems also demonstrated pollen limitation in fruit set. This is the first report of pollen limitation in open-field tomato production in the USA, where previous studies have found no evidence of pollen limitation in hand- versus open-pollinated flowers [11,12].

Field tomatoes were the only crop system that demonstrated significant pollen deficits in both fruit set and weight. The combined effect of these deficits (number of tomatoes harvested × average weight) indicates that yield in the open-pollinated treatment was 35% lower than in the hand-pollinated treatment. Comparing the potential yield loss incurred by insufficient pollination with the yield loss from insect damage, which rarely exceeds 10% in Indiana field tomatoes [47], our results suggest that insect pollination is an under-recognized aspect of tomato production. While managed bumblebees are commonly used in greenhouse settings to supplement tomato pollination, their use in open-field settings is non-existent and would be an interesting avenue for further exploration. In interactions with field tomato growers, they expressed doubt that bees were present in their fields and did not cite pollination as a management concern (author’s personal observation). Thus, the role of pollinators is probably undervalued in field tomato production.

Unlike field tomatoes, open pollination in high tunnels did not improve tomato weight relative to insect exclusion. Supplemental pollination did increase tomato weight, indicating that while gains in weight were possible with increased pollination, the level of insect visitation in high tunnels was insufficient to do so. In our study, results cannot be directly compared between systems because different tomato cultivars were used in each system. Differences in response to insect pollination could, therefore, be attributed to differences in insect visitation and/or cultivars. However, some studies have found fewer bees in high tunnels compared with field settings ([22], author’s unpublished data). In a previous study of the same farms [24], flower observation data from high tunnel tomatoes was excluded due to the very low pollinator abundance recorded (author’s unpublished data). This suggests that (lack of) insect pollination contributes to the difference in pollen limitation observed between field and high tunnel tomatoes, although a study with the same cultivar would be necessary to confirm this.

There are a number of factors that could impede insect pollination in high tunnels. High tunnels reach excessively high temperatures in the summer months, and high temperatures also reduce bee activity, particularly *Bombus* spp. [48], potentially explaining the lack of an increase in fruit weight with insect pollination. Another possibility is that high tunnels containing only tomatoes may not be attractive to a wide range of pollinators. Tomato flower visitor communities in Indiana are dominated by large-bodied bumblebees that are able to extract pollen concealed within the anther cones [24,28]. Unattractive flowers, paired with the additional barriers to entry presented by temperature and the structure of the tunnel, could reduce insect visitation. High tunnels present an area where more research is needed to evaluate the role of insect pollinators, particularly regarding factors affecting their ability to enter and persist in tunnels (e.g. temperature or crop type/diversity).

Field tomatoes were the only crop system that demonstrated evidence of a relationship between pollination treatment and sugar content. Other studies indicate contradictory results, with some reporting no relationship between pollination level and sugar content, and others finding them positively correlated [18,49]. The positive relationship between tomato dry weight and sugar content is one explanation for the observed concurrent increases in weight and Brix for field tomatoes [50]. If so, this implies an additional benefit from insect pollination (and further evidence of pollen limitation), as sugar content is an important quality characteristic for processing tomato varieties [51]. However, while this trend was statistically significant, the change in sugar content (an average absolute increase of 0.14% from open to hand pollination) was small, and thus the agricultural significance of this effect is unclear.

### 4.4. Interannual variability in pollination dynamics

Several crop systems experienced interannual variability in their response to pollination treatments, demonstrating the temporal volatility of pollination dynamics and/or the potential effect of environmental factors. For example, Honeycrisp apples had a lower initial fruit set in 2023 than in 2024 due to a hard frost during bloom. This caused decreased fruit sets in the open and hand pollination treatments, thereby reducing pollen limitation in 2023 compared with 2024. In blueberries in 2024, a cool and overcast period during bloom probably contributed to the lower fruit set and weight recorded in that year by decreasing insect visitation rates [52]. Excessive heat poses a similar problem for summer-blooming crops. High temperatures damage floral organs, decreasing the difference in yield between hand- and open-pollinated flowers (resulting in reduced pollen limitation) while simultaneously lowering the overall yield [53], which is a trend we observed in watermelons. Overall, the interannual variability in yield metrics between and among crops demonstrates how other factors (e.g. weather during bloom) mitigate the benefits of pollination services and extent of pollen limitation.

## 5. Conclusion

In our study of insect pollination across diverse cropping systems, we demonstrate that degree of pollinator dependence and pollen limitation are not always linked. Even in tomato systems, not traditionally considered to be pollinator-dependent or managed with pollination in mind (e.g. not stocked with managed bees), there were enough pollinators persisting in the agricultural landscape to furnish a meaningful difference in yield metrics relative to pollination exclusion, yet pollen limitation persisted. Meanwhile, in more heavily pollinator-dependent apples, thinning negated initial pollen limitation of fruit set. Differences in extent of pollen limitation between crops could be attributed to system-specific factors including management practices, differential pollination requirements and pollinator visitation rates. Additionally, outside factors (e.g. weather during bloom) that affect pollination success and fruit development independent of insect visitation probably contributed to interannual variability in responses. Evaluating pollen limitation in specialty crops is particularly important for identifying systems that could benefit from improvements in pollinator management. For crops that use managed bees, optimized hive management should be considered. According to our results, each crop in our study could benefit to some degree from increasing pollination services, but tomatoes show the most evidence for pollen limitation in multiple yield metrics (fruit set, weight and sugar content). Suboptimal yields may be related to conventional management practices, particularly the heavy use of broad-spectrum insecticides—including neonicotinoids and pyrethroids—that have demonstrated negative effects on pollinating insects. The yield gap incurred by pollen limitation could potentially be addressed by using integrated pest management tools to balance mitigation of pest damage and protection of pollinating insects. Here, we provide benchmark values for specialty crop pollination services and pollen limitation in the Indiana region and the Midwest more broadly, demonstrating the importance of supporting robust pollinator communities to maintain and improve yields in diverse specialty crop systems.

## Data Availability

Data and code are available at https://purr.purdue.edu/projects/indianapollenlimitation/publications. Supplementary material is available online at [54].
